# FEASIBILITY OF A REMOTE SOCIAL NETWORK-BASED OCCUPATIONAL THERAPY PHYSICAL ACTIVITY INTERVENTION

**DOI:** 10.1093/geroni/igae098.3483

**Published:** 2024-12-31

**Authors:** Noah Webster, Margaret Santioni, Lisa Petroff, Maham Adnan, Katrina McGuire, Neil Alexander

**Affiliations:** University of Michigan, Ann Arbor, Michigan, United States; University of Michigan School of Medicine, Ann Arbor, Michigan, United States; University of Michigan, Ann Arbor, Michigan, United States; University of Michigan, Ann Arbor, Michigan, United States; University of Michigan, Ann Arbor, Michigan, United States; University of Michigan, Ann Arbor, Michigan, United States

## Abstract

Despite physical activity being associated with personal and societal benefits, only 16 percent of people age 65 and older engage in recommended levels. To assist in increasing this percentage, we developed a six-week social network-based occupational therapy intervention to be administered remotely with older adults living in subsidized housing. The intervention was designed to improve physical activity through incorporation of socially engaged residents as advisory committee members and addressing activity barriers such as pain through group and individual meetings with an occupational therapist. In summer/fall 2023 a pilot feasibility study was conducted with a cohort of older adults (n=5) living in a Southeast, Michigan housing community. Process outcomes were examined and secondary health and activity outcomes were assessed via pre- and post-intervention interviews and accelerometry. Participants attended on average 9.2 (SD=1.3) intervention activities out of 10. None of the participants knew each other prior to intervention enrollment. However, post-intervention, four of the five participants reported knowing three or more other participants. In terms of secondary outcomes, three out of five participants reported a decline in the extent to which pain interfered with activities and perceived barriers to physical activity decreased for two participants, increased for two, and stayed the same for one. Step counts increased for two participants, but decreased for three. Secondary outcomes were mixed and suggest needed modifications. Additionally, while participation rates exceeded expectations, feasibility was not supported for the social network approach. However, newly formed social ties resulting from the intervention highlight potential for future behavior change.

